# A survey of handling and transportation of UK farmed deer

**DOI:** 10.1017/awf.2023.25

**Published:** 2023-03-14

**Authors:** Samuel J Pearce, Aiden P. Foster, Toby G Knowles, Poppy Statham

**Affiliations:** Bristol Veterinary School, University of Bristol, Langford House, Langford, North Somerset, BS40 5DU, UK

**Keywords:** animal welfare, livestock, meat, red deer, slaughter, transport and handling

## Abstract

Studies on the transport of deer (*Cervidae*), in the UK, were published > 15 years ago. A more recent study of deer transport is required to allow for assessments and improvements to the transport of farmed deer. Sixteen deer farmers participated in a survey describing their management practices related to transport. Their responses showed that most vehicles used to transport deer were designed for other livestock. Participating farmers estimated journey times to slaughter as 1–8 h, with an arithmetic mean of 4.8 (± 2.38) h. Specific concerns raised by the respondents, relating to the transport of deer, included a need for deer-specific vehicles, stop-off areas for long journeys, market locations and haulier experience. Furthermore, data were collected from two abattoirs between July 2019 and June 2020 comprising journey times, slaughter times, bruising, location of origin, vehicle type and the number of animals. In total, 4,922 deer were transported across 133 journeys (from farm to abattoir) from 61 farms. Median and range for journey length were 3.2 (0.4–9.8) h and 154.2 (7.1–462.2) km, whereas group size and time spent in the lairage were 24 (1–121) and 17.8 (10.2–68.9) h, respectively. Group size was found to be significantly associated with both the presence of bruising in a group and the amount of bruising per deer. This study provides a much-needed update on the transport of farmed deer in the UK and highlights key areas for future research including the welfare impact of transport in larger groups and for longer durations.

## Introduction

Transporting livestock is a stressful event that can have a significant impact on animal welfare if performed poorly. Good handling and stockmanship can reduce the stress induced by loading and unloading but the physical and environmental conditions experienced during transport will have a negative effect on all but the most habituated animals. Signs of compromised animal welfare may include behavioural indicators (vocalisation, aggression, higher facial grimace values and a larger amount of visible sclera), physiological responses (increased heart rate and serum cortisol, creatine kinase and lactate dehydrogenase), carcase quality (trauma, pH, dark firm dry meat and pale soft exudative meat), increased disease and mortality. Sheep (*Ovis aries*) that have little experience of being loaded onto vehicles or being transported have been shown to be more negatively affected by the process (Wickham *et al.* 2012). Mixing of social groups is also likely to negatively affect welfare because it risks aggression and fighting, resulting in bruising and reduced product quality due to glycogen depletion (the cause of dark, firm, dry meat). Poor vehicle design, maintenance and road conditions have been associated with an increased incidence of bruising in beef cattle (*Bos taurus*) (Huertas *et al*. 2010). Whilst animals typically adapt to transport over time, long journeys can still result in fatigue, dehydration and hunger if animals are not given sufficient time to rest, drink and eat. This is all further compounded by adverse environmental conditions such as extreme temperatures. It is logical that deer (*Cervidae*) would experience similar, or possibly worse responses due to their more recent domestication compared to that of other livestock species. Capture myopathy is a complication of particular concern in deer (Munro 1994), although most reports are related to the capture of wild deer, it has also been observed in farmed deer (Haigh *et al*. 2005).

The deer farming industry in the UK is relatively small compared to other livestock sectors and has seen a lot of growth over the past couple of decades (Fletcher 2019; Defra 2021). Currently, deer farms are not required to register with a government body in the UK, although the British Deer Farms and Parks Association has reported 89 deer farms as registered members. Deer-specific abattoirs are few in number with two major deer-specific abattoirs operational in mainland Britain today. Anecdotal evidence suggests that some deer are being transported up to 9 h before lairage and slaughter, which may be because of this apparent lack of specialised abattoirs. The maximum journey length reported in the scientific literature is 6 h for experimental groups of six animals (Grigor *et al*. 1998c), whereas commercial deer farms are likely to transport much larger groups of animals and therefore it is unclear how translatable those findings are to the transport of commercial farmed deer today.

Regarding pre-transport handling, the European Food Safety Authority (EFSA) has recommended that raceways for loading red deer (*Cervus elaphus*) should be at least 5 m in width (EFSA 2004). This recommendation contrasts with other reports on pre-transport loading of deer that found the animals more easily entered races 1.5-m wide and were subsequently loaded onto the trailer faster; compared to 4- or 0.5-m wide races, likely because deer could less easily turn around and run behind handlers in 4-m wide races and that they prefer to move as a group rather than single file, as in 0.5-m wide races (Grigor *et al*. 1998b). EFSA also recommended that ramps should be avoided if practical, or up to 10° to minimise risks if the deer were to panic. However, they did note that deer can climb steeper ramps if they have cleats (EFSA 2004), yet there seems to be little research to support this recommendation with the exception of an anecdotal observation in one study (Smith & Dobson 1990).

There have been several studies on specific welfare measures of deer during transport; however, most were performed 20 to 25 years ago and involved predominantly small, experimental groups of animals. It has been demonstrated that while transport is stressful for deer, it is difficult to determine if longer journeys are increasingly stressful as different measures of physiological stress are not consistent with that hypothesis. For example, heart rate and lactate have been reported to increase greatly at the beginning of transport, then quickly recover, suggesting that the deer had become accustomed to the transport (Waas *et al*. 1997; Grigor *et al*. 1998c). On the other hand, plasma cortisol and sodium appeared to increase linearly with journey length (Waas *et al*. 1997), suggesting that deer became increasingly stressed over time. Furthermore, significantly more bruising was reported on the hock, hindquarter and back of deer after longer journeys, whereas overall carcase bruise score tended to be higher with longer journeys but not significantly so (*P* = 0.09) (Jago *et al*. 1997).

The Farm Animal Welfare Committee (FAWC) suggested that overnight lairage of deer may be suitable prior to slaughter (FAWC 2013). Generally, the aforementioned studies do support the concept that while deer are physiologically stressed during transport, they will recover, to a large extent, if given time to rest afterwards (Jago *et al*. 1997; Grigor *et al*. 1998a,b; Waas *et al*. 1999). However, Pollard *et al*. (2002) observed significant differences between plasma and serum biochemistry values in deer shot in the field and those slaughtered at an abattoir which suggested that deer shot in the paddock were less stressed at the time of death, despite the deer slaughtered at an abattoir being held in lairage overnight, suggesting that the increased biochemical values observed and animal stress occurred during lairage or at the time of slaughter. Further, deer appear to take longer to become accustomed behaviourally to their new surroundings. One study reported that deer only began to lie down after 3 h in lairage and it took up to 10 h for the proportion of deer lying down to reach near pre-transport levels (Grigor *et al*. 1997). Alongside this, aggressive behaviour was seen to increase among deer after spending 8 h in lairage and liveweight decreased over time, although this appeared to have little effect on hot carcase weight (Grigor *et al*. 1997).

The evolution of the deer industry since the 1990s, combined with the lack of information regarding the management of commercial deer around transport and the current methods of deer transport and relevant management, makes it unclear how translatable the previous studies are to the commercial deer industry today and highlights the need for more up-to-date information to inform the assessment of the welfare of deer during transport in the UK.

This study aimed to gather information on the handling and management of farmed deer prior to and during transport; to quantify and describe aspects of transport to slaughter and investigate whether they are associated with carcase bruising, by surveying deer farmers and analysing data collected from abattoirs handling deer. The aim of this study was to highlight areas of deer transport that could be improved for the benefit of deer welfare and carcase quality, or areas that require further investigation to determine the impact on deer welfare.

## Materials and methods

A survey (see Appendix 1) was designed online using Microsoft Forms® (Microsoft®) and was distributed to British deer farmers in newsletters from the British Deer Farms and Parks Association and the British Deer Society. A deer-dedicated abattoir also circulated the survey information to their customers. The survey was expected to take 10 min to complete and aimed to collect data relating to the farm itself, including the deer species kept, purpose of the deer herd, tameness of the deer and each farm’s handling facilities. The survey also asked questions on if and how the farm transported deer by collecting data on pre-movement management changes, vehicle types, purposes of travel, group sizes, journey length, feed and water supply around transport, ramp angles and races for each of the journey types. Journey types were defined as local journeys (< 1 h), long journeys (> 1 h) and transport to slaughter. Journeys to slaughter were not mutually exclusive from the other types of journeys. Finally, the questionnaire asked about transport of deer in hard antler, concerns that farmers may have about deer transport and the effect of COVID-19 on their business.

Transport data were collected retrospectively from the two abattoirs in the UK that handle deer, relating to all the deer slaughtered between July 2019 and June 2020. The data comprised journey dates, loading and unloading times, group size, vehicle types, location transported from, time or date slaughtered, weight of condemned meat due to bruising and whether any whole carcases were condemned.

### Statistical analysis

Descriptive statistics were produced using R (Version 3.6.3) (R Core Team 2020) and RStudio (Version 1.3.959) (RStudio Team 2020). Distances between farms and the respective abattoir were calculated (in km) as a direct, straight line between the two locations. This was not the actual route taken, but chosen as a standardised and simple proxy for journey distance, as a number of variables could not be controlled for, such as road access on the day of transport (due to terrain, weather, roadworks, vehicle size and traffic flow/congestion, as recommended by a search engine such as Google Maps), potential for multiple pick-ups and journey breaks. Average weight of carcase meat condemned per deer was calculated based on the total weight of condemned meat in the group and the number of deer in the group. The mean and standard deviation, or median and IQR (where data were skewed) were calculated for each variable. Ranges are provided. Binary logistic regression was used to determine associations between journey time and the natural log of group size with the presence of bruising in a group; a general linear model was used to assess statistical associations between journey time and the natural log of group size with the weight of carcase meat condemned due to bruising per deer in the group. Tjur’s R^2^ and R^2^ were calculated for the binary logistic regression and general linear model, respectively.

### Ethical approval

This study received ethical approval from the University of Bristol Research Ethics Committee. Survey participants were provided with a participant information sheet (Appendix 2) discussing the purpose and aims of the study and were required to electronically sign a consent form before beginning the survey which stated the purpose and use of the collected data (Appendix 1). All data were collected anonymously and participants were given the option to revoke their consent at any time.

## Results

### Survey

**Figure 1.**
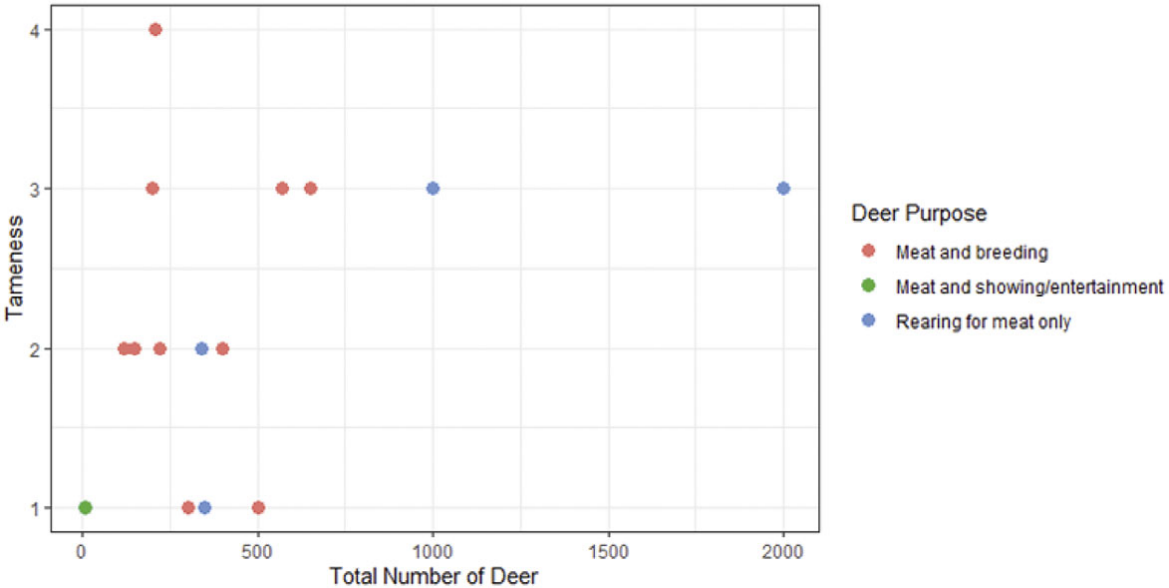
Farmer reported tameness of their deer against the total number of deer and their purpose. Tameness was reported on a scale of 1-5 where 1 was very tame and the deer will come within arm’s length; and 5 was flighty and will move away at the sight of people.

Sixteen deer herd owners responded to the survey, all of which farmed red deer, with one farm also keeping fallow deer (*Dama dama*). All farms reared their deer for meat, with ten also keeping breeding stock and two keeping deer for showing or entertainment purposes. The median number of deer on each farm was 320 (range: 10–2,000; IQR = 330) and when asked to rate how tame their deer were on a scale of 1 to 5 (1 = very tame and will come within an arm’s length; 5 = flighty and will run away at the sight of people), the mean was 2.1 (± 0.96) (range = 1–4) (Figure 1).

Alternatives to transporting to slaughter were used on seven farms, five shooting deer in the field and two using an on-site abattoir; this question did not preclude the use of a commercial abattoir in conjunction with these methods. Two respondents also reported transporting deer in hard antler.

Nine farmers transported deer for local journeys. Of these, four separated the deer into housing or a pen next to the herd, two housed or penned them away from the herd and three did not separate them at all prior to transport. Out of 12 respondents that said they transported deer over long distances, seven housed or penned them next to the herd, five housed or penned them away from the herd and seven said that they specifically provided food and water prior to long journeys. Of the eight that transported deer to slaughter, four housed or penned the deer next to the herd and four away from the herd. Thirteen respondents reported removing antlers prior to travel, with removal seven days in advance of transport being the minimum, although many respondents reported doing this multiple weeks in advance; up to two months. One respondent removed antlers from all stags in September. All who removed hard antlers restrained the deer in either a hydraulic, squeeze or drop floor crush.

Fourteen respondents had a purpose-built crush, with 12 of these having a purpose-built race as well and one of these having both a purpose-built race and a mobile crush. One farmer had a mobile crush with both a purpose-built race and a mobile race and the final respondent only reported having a mobile race. Reported race widths varied from 1.5 m (n = 1), 2 m (n = 5), 2.5 m (n = 3) up to a maximum of 3 m (n = 2). Ramp angles differed for transport to slaughter, which varied from no ramp at all (n = 1), 10° (n = 2), 20° (n = 4) and up to 30° (n = 2), compared to that of local and long journeys which included ramps of 10° (n = 1), 20° (n = 5) and 30° (n = 2), although a further five farmers did not know the ramp angles that they used. The majority of deer groups were not mixed for transport, however two farmers indicated that they mixed groups for local transport and one for long journeys.

Unsurprisingly, all within-farm movements were short journeys (< 1 h), however, half of the temporary movements to other farms were classed as long journeys, as were just under half of permanent moves to other farms and the majority of journeys to slaughter.

According to the respondents, local and long transports had median journey times of 20 min (range: 10–60 min; IQR = 15 min) and 5 h (range: 2–20 h; IQR = 3.25 h), respectively. Transport to slaughter had a mean journey time of 4.8 (± 2.38) h (range: 1–8 h). Data presented in Table 1 would suggest that these longer journeys tended to contain larger groups of deer. Only one respondent said that they exported deer internationally and that they had previously sent deer to France and Denmark, although this was several years ago.

**Table 1.** Survey results for deer group size, vehicle details and driver information across local journeys, long journeys and transport to slaughter

Characteristic	Local journeys (n = 9)	Long journeys (n = 12)	Journeys to slaughter (n = 8)
***Group size***			
< 10	3 (33.3%)	1 (8.3%)	0
10–30	4 (44.4%)	5 (41.7%)	4 (50%)
> 30	2 (22.2%)	6 (50%)	4 (50%)
***Vehicle decks***			
Single	7 (77.8%)	4 (33.3%)	4 (50%)
Double	1 (11.1%)	7 (58.3%)	4 (50%)
Varies	1 (11.1%)	1(8.3%)	0
***Vehicle design***			
Designed for deer	3 (33.3%)	3 (25%)	1 (12.5%)
Designed for other livestock	6 (66.7%)	9 (75%)	6 (75%)
Both	0	0	1 (12.5%)
***Vehicle type***			
Car/tractor and trailer	9 (100%)	4 (33.3%)	1 (12.5%)
Fixed bed/rigid lorry	0	4 (33.3%)	4 (50%)
Articulated lorry	0	7 (58.3%)	5 (62.5%)
***Transport driver***			
Farmer/employee	9 (100%)	3 (25%)	1 (12.5%)
Haulier	0	9 (75%)	7 (87.5%)

Finally, the question of whether any aspects of deer transport were deemed to have scope for improvement saw comments made regarding the need for deer-specific lorries and stop-off areas when transporting deer over long distances, having a market closer to the farm and the variability of farmer handling facilities, as well as haulier facilities and experience. A number of respondents said that their business was affected by COVID-19, with the exception of one that had to stop offering veterinary student placements and another that had been considerably affected because they supplied meat to restaurants and the closure of these meant impacted demand, meaning less deer than expected being slaughtered and sold.

### Transport to slaughter results

**Figure 2.**
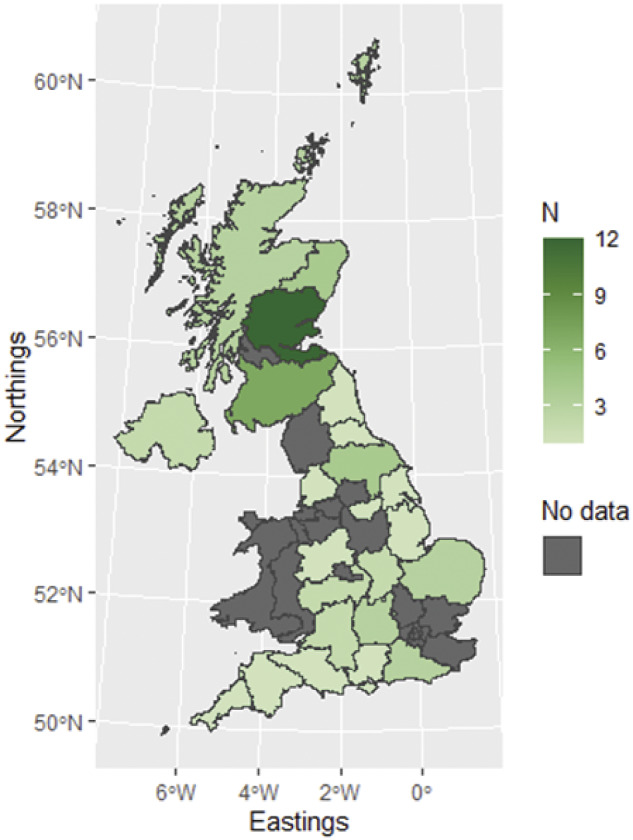
UK county heatmap of the location of deer farms supplying participating abattoirs.

A total of 4,922 deer were transported across 133 journeys from 61 farms (see Figure 2 for farm distribution) in the 12-month period.

**Figure 3.**
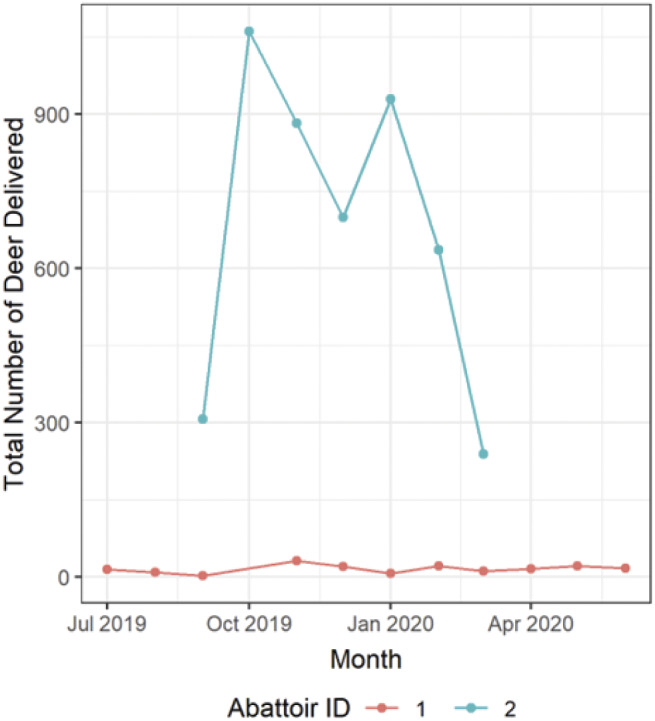
Number of deer being delivered to the two participating abattoirs by month.

Abattoir 1 processed small numbers of deer throughout the year and whilst abattoir 2 only operated from September to March, they processed 96.6% of the deer from 78.2% of all vehicles in this study, with the largest demand over the October–January period (Figure 3). Group sizes varied greatly throughout the year (median: 24; range: 1–121; IQR = 49).

**Figure 4.**
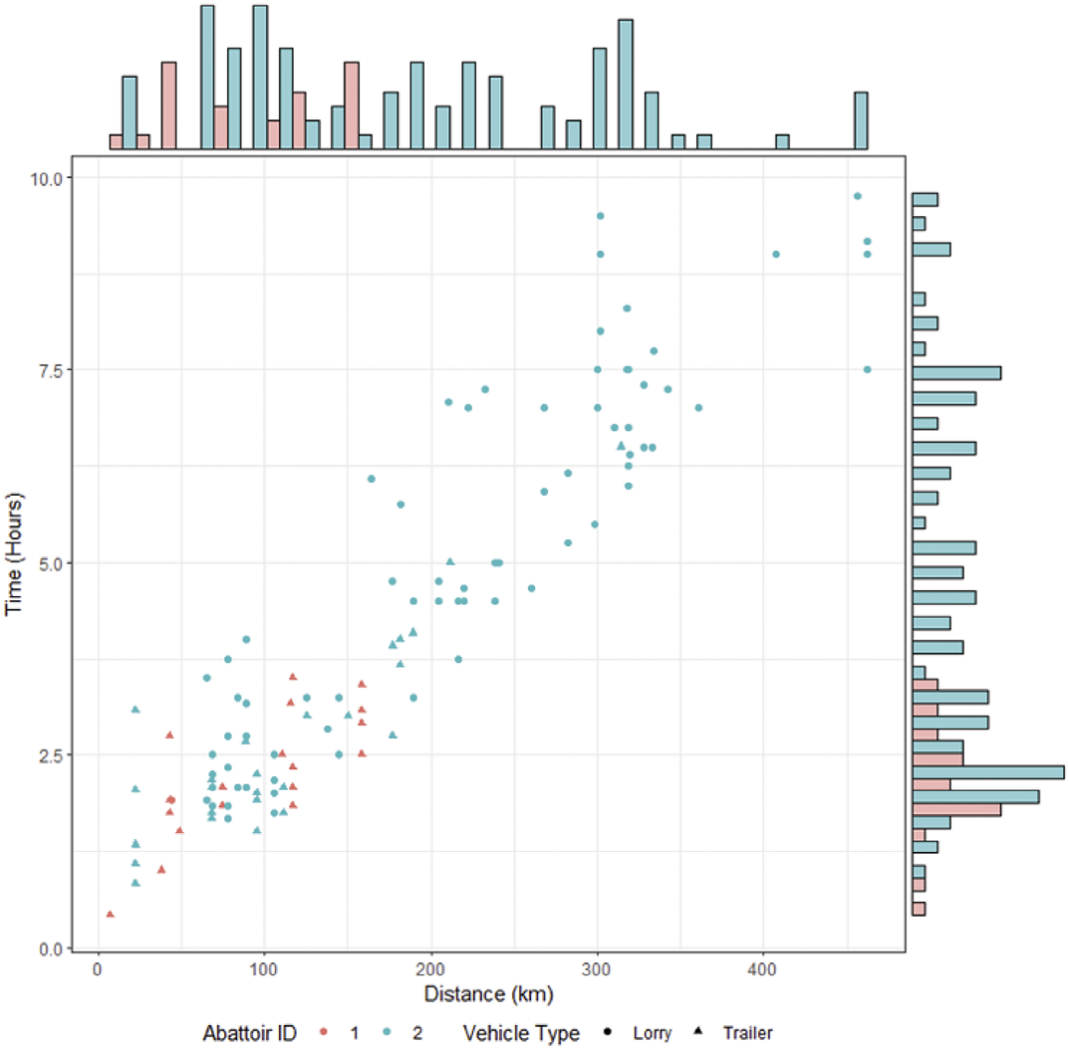
Time against distance with associated histograms for the journeys of groups of deer, by both vehicle type and abattoir identification.

**Figure 5.**
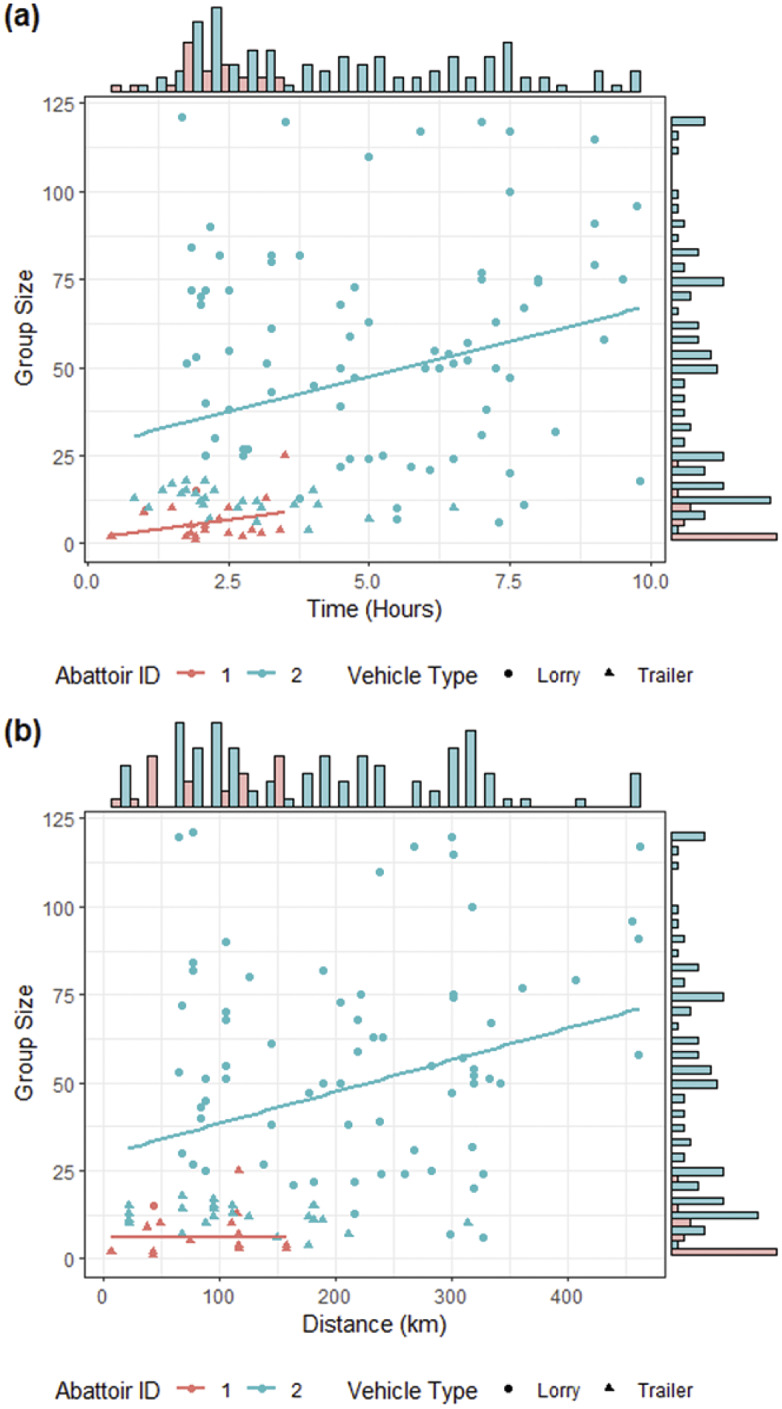
Group size against (a) length of journey time and (b) distance for journeys of groups of deer to two abattoirs (1 and 2) and by vehicle type (with marginal histograms).

Deer were generally loaded into vehicles in mid-morning and unloaded in early afternoon. Although shorter journeys were more common, abattoir 2 (the abattoir receiving more deer) had much more variability in the length of time for each journey, with some journeys taking as much as three times longer than the maximum journey length for the smaller abattoir (Figure 4). In addition, very few of the abattoir-recorded journeys were less than 1 h aligning with our survey results which found that few local journeys were to slaughter. The median values for journey length and distance between the farm and abattoir were 3.2 h (range: 0.4–9.8; IQR: 4.0) and 154.2 km (range: 7.1–462.2; IQR: 179.9), respectively. Data presented in Figure 5 would suggest that larger groups were being transported from farms further away and for longer periods of time, although some small groups may be transported by trailer for up to 7.5 h, from farms up to 314 km away. Further, Figure 5 also shows that larger groups were generally transported in livestock lorries, with trailers only being used for groups of 25 or less animals.

**Figure 6.**
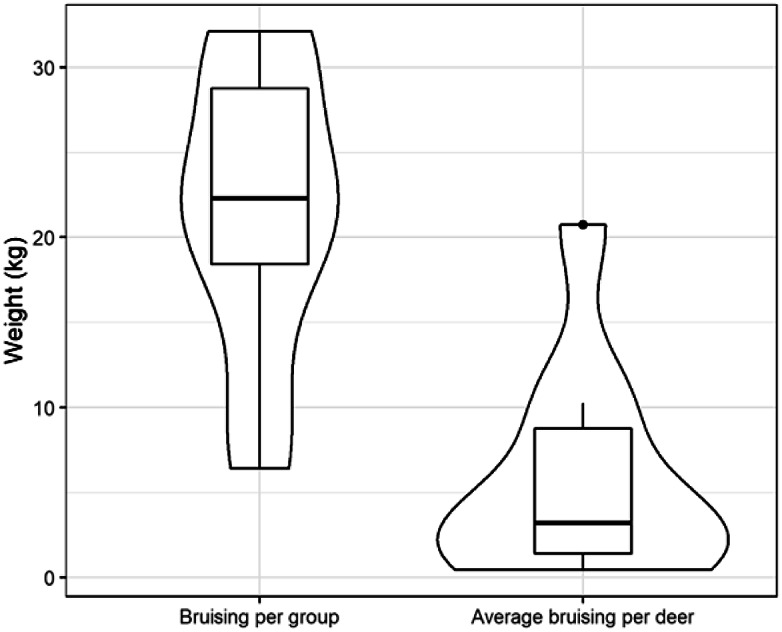
Boxplot of the total weight (kg) of carcase material condemned per group and the average weight condemned per deer in the group across both abattoirs. For comparison the dressed carcase weight for red deer hinds and stags at 15-18 months of age is 45-55 kg.

Once at the abattoir, median lairage time was 17.8 h (range: 10.2–68.9; IQR; 2.95). Bruising was recorded in ten of 133 (7.5%) journeys comprising a total of 128 deer, accounting for a total of 215.6 kg of meat being condemned; no whole carcases were condemned. In those groups that did have meat condemned, the mean weight was 21.6 (± 8.63) kg per group (range: 6.4–32.1) and when taking group size into account the average weight condemned per deer was 5.6 kg (range: 0.5–20.7; IQR: 7.4). For comparison dressed red deer carcases are of the order of 45 and 55 kg, for hinds and stags, respectively, at 15–18 months old, so the average amount of carcase that was condemned constituted 5.6/55 or about 10% of the carcase (see Figure 6).

There was a negative relationship between group size and the presence of bruising (*P* < 0.05; OR: 0.33) (Table 2) and the amount of meat condemned per deer in a group (*P* < 0.05), with an estimated decrease of 7.15 kg in meat per animal for each increase in the natural log of group size (Table 3).

**Table 2.** Binary logistic regression results for the presence of bruising within a group

	Presence of bruising in the group		
Predictors	Odds ratio	Confidence interval	*p*
Intercept	1.64	0.29–10.37	0.577
In (number of deer)	0.33	0.14–0.68	**0.005**
Journey time	0.91	0.51–1.42	0.698

Observations n = 127; R^2^ Tjur = 0.179.

*P*-values < 0.05 were considered to be statistically significant; values < 0.05 are highlighted in bold.

**Table 3.** Linear regression results for the weight of meat condemned for bruising within a group

	Bruising per deer in group (kg)		
Predictors	Estimates	Confidence interval	*P-*value
Intercept	7.14	–1.46–15.75	0.089
Journey time	4.17	–1.04–9.37	0.098
In (number of deer)	–7.15	–12.28–2.01	**0.014**

Observations n = 9; R^2^ / R^2^ adjusted = 0.718/0.624.

*P*-values < 0.05 were considered to be statistically significant; values < 0.05 are highlighted in bold.

## Discussion

In 2020, Defra estimated that there were 37,000 farmed deer in the UK (Defra 2021) and the total number of deer held by survey respondents was 7,030, therefore our survey covered about 19% of the UK farmed deer population. The responses highlighted that farmed deer are almost exclusively red deer, with farm populations appearing to vary considerably in size and tameness. Many farms employed other on-site slaughtering methods, such as an on-site abattoir or shooting in the field, although it was not specified how these animals entered the food chain.

Most of the surveyed farms removed hard antlers from stags multiple weeks in advance of transport, in line with Defra recommendations (Defra 1989). Those that removed antlers seven days before transport may find increased carcase bruising if taken to slaughter immediately (Goddard 1994), however we did not investigate any association between antler removal and carcase bruising or meat condemnation in this study. Furthermore, the effect of transport on welfare after antler removal is unclear because carcase bruising occurs due to restraint during antler removal. Two farms indicated that they transported deer in hard antler. This may pose some welfare problems because deer in antler are required to be transported in their own pen and we have shown here that small group sizes were associated with larger amounts of meat condemned per deer.

It appeared to be common for races to be 2–3-m wide and ramp angles to be > 10°. The authors did not find any published studies on the safety or aversiveness of different ramp angles for deer. While many farmers surveyed here used ramps greater than the recommended 10° (EFSA 2004) and may find these suitable depending on the temperament of their deer, further studies are required.

Regarding the transport itself, some farmers reported mixing deer groups which is a potential source of stress for the animals (EFSA 2004). General trends relating to the vehicles included the migration towards larger vehicle types and the use of hauliers with longer journeys and journeys to slaughter. This may be linked to the findings that these types of journeys involved larger groups of deer and the finding that most long journeys were transport to slaughter. Whilst deer farms supplying the two participating abattoirs were concentrated in central and southern Scotland, there were deer farms across the whole country, including as far south as Cornwall and two farms in Northern Ireland. Considering that the two abattoirs are located on the UK mainland, deer are presumably ferried to the mainland before being driven for the remainder of the journey. As far as the authors are aware, there are no studies on the welfare of deer during transport over water.

In this study, the journey lengths and groups sizes showed vast ranges and were much longer and larger than those reported in previous studies. For example, Grigor *et al*. (1998a) investigated the behavioural and physiological effects of group size using groups of five or ten deer and Waas *et al.* (1997) used groups of six deer. However, larger groups transported in lorries or large trailers are likely to be split between several pens, with a level of detail not able to be estimated with our data collection method. Regarding journey length, our study showed that deer were transported for up to a maximum of 9.8 h or from farms up to 462.3 km away and there are no previous studies investigating journey lengths more than 6 h (Grigor *et al*. 1998c) or 380 km (Jago *et al*. 1997).

Group size was significantly associated with the presence of carcase meat being condemned, with a decreased odds of meat condemnation being present within a group and group size was negatively correlated with the amount of meat being condemned per deer in the group. One possible explanation for this would be the increased use of livestock lorries with larger group sizes, although further studies would be needed to confirm any association with vehicle types. Since the data were collected retrospectively and the scoring not standardised between abattoirs, there are a number of potential explanations for the difference in the amount of meat being condemned at each abattoir. We were also unable to rule out other links to bruising such as the time spent in lairage, the abattoir used and stocking density. Although it was not statistically significant, there was a tendency for journey length to be correlated with the amount of bruising per deer which is similar to previous findings (Jago *et al.* 1997). As the population of deer with bruising was small, a larger controlled study would be needed to attempt to elucidate any relationship between journey time and bruising or meat condemnation.

Farmers highlighted a number of concerns about the need for deer-specific vehicles, a recommendation echoed by EFSA (2004), as well as the need for closer markets and areas to stop off on longer journeys. Unfortunately, it is unclear how important reducing these transport times are for deer welfare, because any association between journey length and bruising was not significant and as previously mentioned, different physiological measures of stress have had contrasting results as to whether deer are increasingly stressed over time, or become accustomed to the transport (Jago *et al*. 1997; Waas *et al*. 1997; Grigor *et al*. 1998a).

The transport data present several limitations because they do not contain information on rest stops made by transporters, whether the same vehicle was used for multiple pick-ups or if and how animals were separated between different pens in the vehicles. In addition, the distance was a proxy measure using the distance between the farm and abattoir which may not represent the distance actually covered by the vehicle. These data have been included as estimates for descriptive purposes but should be interpreted carefully, taking into account the method of calculation. The criteria for meat condemned due to bruising was also not specified and therefore may not be consistent between abattoirs. In addition, bruises are difficult to age and have previously been difficult to associate with traumatic events (Strappini *et al*. 2009, 2012; Kline *et al*. 2020). Therefore, it is possible that the bruising reported may have occurred pre- or post-transport and significant results should be interpreted with caution.

### Animal welfare implications

This study provides a much-needed update on how deer are being transported in the UK, highlighting that journeys for deer can be over long distances and durations. Given that one of the two abattoirs has since closed, journey lengths may have already increased further. Amongst the deer farmers surveyed concern was expressed at a lack of both suitable stop-off locations and availability of deer-specific transport, indicating that stopping may be difficult and that many journeys are undertaken in vehicles designed for other livestock species. Controlled studies measuring welfare would be needed to confirm whether these have welfare implications.

In addition, the abattoir data demonstrated that smaller groups of deer were more likely to be associated with bruising during transport. A controlled study would be required to confirm the causal factors in this relationship, but this variation indicates potential areas for improving welfare. The survey results indicated considerable variation in handling facilities and pre-transport management giving further areas to explore with potential to improve the welfare of transported deer.

## Conclusion

In summary, this preliminary study used a survey of deer farmers and data collected at deer abattoirs, to give an updated picture of deer transport in the UK. Three-quarters of farmers surveyed transported their deer over long distances and the abattoir data confirmed that these include larger groups and longer distances than was found in previous research studies. Deer farmers themselves raised concerns regarding a lack of deer-specific transport and lack of stop-off locations for these long journeys. This highlights a need for controlled studies on the impact of long journeys especially in non-specialised vehicles on both meat quality and deer welfare. The results of the survey also indicated considerable variation in the handling facilities and management on farms including the width of races, angles of ramps, the timing of antler removal (or lack of) and occurrence of mixing. Further research is needed to understand the impact these have on deer welfare.

## Supplementary material

For supplementary material accompanying this paper visit https://doi.org/10.1017/awf.2023.25. click here to view supplementary material
