# Supplementary material for: A survey of handling and transportation of UK farmed deer
Source: Anim Welf. 2023 Mar 14;32:e30. doi: 10.1017/awf.2023.25 (PMC10936360; doi:10.1017/awf.2023.25)
Supplement: Supplementary file 1 [file awfsup.zip › S0962728623000258sup001.pdf]

An investigation into the current transportation methods used for captive UK deer

Thank you for your interest in our survey. As someone that works with deer, your responses will help us gain a better understanding of the transport of deer in the UK. This will help identify areas for improvements that may benefit you and the welfare of the deer. This survey should take around 10 minutes to complete.

We apologise for repetition of certain questions, but this is the only way we can capture the relevant information and keep the survey easy to follow.

When answering these questions, please answer them based on your experiences during a normal year prior to the Coronavirus pandemic. There is room at the end to discuss how the Coronavirus has effected your work.

Please read the prepared participant information sheet created to help you understand the purpose and aims of this study, as well as, what is involved in this survey:
https://uob-my.sharepoint.com/:b:/g/personal/sp16767_bristol_ac_uk/EbpHf8Nql4xBqVehQzAgxAABtX27wSEIgtOyJ1ISCjNctg (https://uob-my.sharepoint.com/:b:/g/personal/sp16767_bristol_ac_uk/EbpHf8Nql4xBqVehQzAgxAABtX27wSEIgtOyJ1ISCjNctg)

This research has been approved by the University of Bristol Research Ethics Committee. If you do have any questions regarding this survey or the rest of the study, please do not hesitate to get in contact with Samuel Pearce on the email below.

Samuel Pearce, BVSc Veterinary Science Student, University of Bristol, sp16767@bristol.ac.uk (mailto:sp16767@bristol.ac.uk).
Dr Poppy Statham, Network Manager for Animal Welfare Research Network, University of Bristol.
Prof. Toby Knowles, Professor of Farming and Food Science, University of Bristol.
Dr Aiden Foster, Senior Lecturer in Veterinary Dermatology and Pathology at the University of Bristol and President of the Veterinary Deer Society.

* Required

Consent

1. Have you been given information explaining the study? *

◯ Yes

◯ No

2. Have you had an opportunity to ask questions and discuss this study? *

◯ Yes

◯ No

3. Have you received satisfactory answers to all questions you have asked? *

◯ Yes

◯ No

◯ N/a - Did not ask any questions

4. Have you received enough information about the study for you to make a decision about your participation? *

◯ Yes

◯ No

5. I understand that my participation is voluntary and that I am free to withdraw my information prior to publication at any time and without giving a reason. *

◯ Yes

◯ No

6. I hereby fully and freely consent to my participation in this study *

By ticking below to provide my consent, I confirm that:

I understand the nature and purpose of the procedures involved in this study. These have been communicated to me on the information sheet accompanying this form.
I understand and acknowledge that the investigation is designed to promote scientific knowledge and that the University of Bristol will use the data I provide for no purpose other than research.
I understand the data I provide will be kept confidential. My name or other identifying information will not be disclosed in any presentation or publication of the research.
I understand that the University of Bristol may use the data collected for this project in a future research project but that the conditions on this form under which I have provided the data will still apply.

◯ I give my consent to take part in this study.

Premise Information

7. What type of deer holding is your premise? *

- ◯ Farm
- ◯ Park
- ◯ Zoo
- ◯ Personal Holding
- ◯ Other

8. Please provide the first half of your postcode *

E.g: BS45

This will only be used to assess differences in responses based on geographic location. We have only asked for the first half of the postcode to ensure that the information is not personally identifiable.

9. What species of deer do you keep? *

Tick all that apply.

- ☐ Red
- ☐ Fallow
- ☐ Reindeer
- ☐ Other

10. How many deer do you keep in total? *

The value must be a number

11. What are your deer kept for? *

Tick all that apply

- [ ] Reared for meat
- [ ] Breeding stock
- [ ] Showing/entertainment purposes (e.g. zoo)
- [ ] Personal interest/pets
- [ ] Other

12. In general, how tame are your deer? *

1 = Very Tame - Deer will happily be within arms length and may even tolerate contact.
5 = Not tame/Flighty - Deer will run away at the sight of people

	1	2	3	4	5	
Very Tame	○	○	○	○	○	Not Tame/Very Flighty

13. What handling/loading facilities do you use? *

Tick all that apply

- [ ] Mobile Race
- [ ] Mobile Crush
- [ ] Purpose-built race
- [ ] Purpose-built crush
- [ ] None

Transport of deer locally

E.g. Transport within your holding or journeys under 1 hour.

14. Do you transport deer locally? *

Local transport: Transport within your holding or journeys under 1 hour.

- ◯ Yes
- ◯ No

15. What pre-movement management changes do you use? *

For Local transport: Transport within your holding or journeys under 1 hour.

Tick all that apply

- ☐ Penned separately next to herd
- ☐ Housed/penned away from the herd
- ☐ None
- ☐ Other

16. Who is normally driving the transport? *

For Local transport: Transport within your holding or journeys under 1 hour.

- ◯ Yourself or other employee
- ◯ Hired transporter/haulier
- ◯ Other

17. What type of transport vehicles would be normally used for local travel? *

For Local transport: Transport within your holding or journeys under 1 hour.

Tick all that apply

- ☐ Car & Trailer
- ☐ Articulated lorry
- ☐ Fixed bed/rigid lorry
- ☐ Other

18. Is this vehicle purpose built for deer, or originally designed for use with other livestock? *

For Local transport: Transport within your holding or journeys under 1 hour.

- ○ Designed for deer
- ○ Designed for other livestock
- ○ Other

19. Is this vehicle normally single or double deck? *

For Local transport: Transport within your holding or journeys under 1 hour.

- ○ Single
- ○ Double
- ○ Varies

20. What are the purposes for this travel? *

For Local transport: Transport within your holding or journeys under 1 hour.

Tick all that apply

- [ ] Within-farm movement
- [ ] Temporary movement to other farms (i.e. breeding purposes)
- [ ] Permanent movement to another farm (i.e. sold stock)
- [ ] Transport to slaughter
- [ ] Other

21. What is the average total group size for these local journeys? *

For Local transport: Transport within your holding or journeys under 1 hour.

- ◯ <10
- ◯ 10-30
- ◯ 30+

22. Generally, are separate groups of deer mixed to be transported? *

For Local transport: Transport within your holding or journeys under 1 hour.

- ◯ Yes
- ◯ No
- ◯ Other

23. On average, how long are these local journeys (minutes)?

For Local transport: Transport within your holding or journeys under 1 hour.

The value must be a number

24. What is the maximum ramp angle on these vehicles for loading/unloading deer?

For Local transport: Transport within your holding or journeys under 1 hour.

- 10
- 20
- 30
- No ramps
- Don't know
- Other

25. If you use a race, how wide is the race used to load deer onto the vehicle (in metres)?

For Local transport: Transport within your holding or journeys under 1 hour.

The value must be a number

Transport of deer over long distances

E.g. Journeys over 1 hour

26. Do you transport your deer over long distances?

For Long Distance transport: Journeys over 1 hour

- ◯ Yes
- ◯ No

27. What pre-movement management changes do you use?

For Long Distance transport: Journeys over 1 hour

Tick all that apply

- ☐ Housed/Penned next to herd
- ☐ Housed/Penned away from the herd
- ☐ None
- ☐ ____________________

  Other

28. Who is normally driving the transport? *

For Long Distance transport: Journeys over 1 hour

- ◯ Yourself or other employee
- ◯ Hired transporter/haulier
- ◯ ____________________

  Other

29. What type of transport vehicles would be normally used for long distance travel? *

For Long Distance transport: Journeys over 1 hour

Tick all that apply

- [ ] Car & Trailer
- [ ] Articulated lorry
- [ ] Fixed bed/rigid lorry
- [ ] Other

30. Is the vehicle purpose built for deer, or originally designed for use with other livestock? *

For Long Distance transport: Journeys over 1 hour

- ◯ Designed for deer
- ◯ Designed for other livestock
- ◯ Other

31. Is this vehicle normally single or double deck? *

For Long Distance transport: Journeys over 1 hour

- ◯ Single
- ◯ Double
- ◯ Varies

32. What are the purposes for this travel?

For Long Distance transport: Journeys over 1 hour

Tick all that apply

- [ ] Temporary movement to other farms (i.e. breeding purposes)
- [ ] Permanent movement to another farm (i.e. sold stock)
- [ ] Transport to slaughter
- [ ] Export of livestock to another country
- [ ] Other

33. If you export live deer to other countries, please list the countries that you have sent deer to.

For Long Distance transport: Journeys over 1 hour.

34. What is the average total group size for these long journeys? *

For Long Distance transport: Journeys over 1 hour

- ○ <10
- ○ 10-30
- ○ 30+

35. Generally, are separate groups of deer mixed to be transported? *

For Long Distance transport: Journeys over 1 hour

- ○ Yes
- ○ No
- ○ Other

36. On average, how long are these long distance journeys (hours)?

For Long Distance transport: Journeys over 1 hour.

[ ]

The value must be a number

37. What is the maximum journey length your deer have been transported (hours)?

For Long Distance transport: Journeys over 1 hour.

[ ]

The value must be a number

38. Do you supply or withdraw feed and water before long journeys?

For Long Distance transport: Journeys over 1 hour.

If this depends on the distance/length of time, please tick the appropriate box(es) and use the "other" box to type the minimum journey length that you would consider giving/withdrawing food on.

- [ ] Give food and water specifically before a long journey
- [ ] Withdraw food and water specifically before a long journey
- [ ] Neither
- [ ] Other

39. Do you supply feed and water during long journeys? *

For Long Distance transport: Journeys over 1 hour.

If this depends on the distance/length of time, please tick yes and use the "other" box to type the minimum journey length that you would consider giving feed and water on.

- ☐ Yes
- ☐ No
- ☐ Other: ______

40. What is the maximum ramp angle on these vehicles for loading/unloading deer?

For Long Distance transport: Journeys over 1 hour.

- ○ 10
- ○ 20
- ○ 30
- ○ No ramps
- ○ Don't know
- ○ Other: ______

41. If you use a race, how wide is the race used to load deer onto the vehicle (in metres)?

For Long Distance transport: Journeys over 1 hour.

______

The value must be a number

Transport of deer to slaughter

42. Do you slaughter deer via methods other than transport to an abattoir? *

Tick all that apply

- [ ] Shooting in the field
- [ ] On-site abattoir
- [ ] No other methods
- [ ] ______

Other

43. Do you transport deer to abattoirs? *

- ( ) Yes
- ( ) No

44. What abattoir do you use?

______

45. What pre-movement management changes do you use?

For transport to an abattoir.

Tick all that apply

- [ ] Housed/Penned next to herd
- [ ] Housed/penned away from herd
- [ ] none
- [ ] ______

Other

46. Who is normally driving the transport?

For transport to an abattoir.

- ◯ Yourself or other employee.
- ◯ Hired transporter/haulier
- ◯ Other

47. What type of transport vehicles would be normally used for transport to slaughter?

For transport to an abattoir.

Tick all that apply

- ☐ Car & trailer
- ☐ Articulated lorry
- ☐ Fixed bed/rigid lorry
- ☐ Other

48. Is the vehicle purpose built for deer, or originally designed for use with other livestock? *

For transport to an abattoir.

- ◯ Designed for deer
- ◯ Designed for other livestock
- ◯ Other

49. Is this vehicles normally single or double deck? *

For transport to an abattoir.

- ◯ Single
- ◯ Double
- ◯ Varies

50. What is the average total group size for these journeys to an abattoir? *

For transport to an abattoir.

- ◯ <10
- ◯ 10-30
- ◯ 30+

51. Generally, are separate groups of deer mixed to be transported? *

For transport to an abattoir.

- ◯ Yes
- ◯ No
- ◯ Other

52. How long does this journey take on average (hours)?

For transport to an abattoir.

The value must be a number

53. What is the maximum ramp angle on these vehicles for loading/unloading deer?

For transport to an abattoir.

- ◯ 10
- ◯ 20
- ◯ 30
- ◯ No ramps
- ◯ Don't know
- ◯ Other: ____________

54. If you use a race, how wide is the race used to load deer onto the vehicle (in metres)?

For transport to an abattoir.

____

The value must be a number

General Transport Information (any distance/destination)

55. Do you ever transport stags in hard antler?

- ◯ Yes
- ◯ No

56. If you remove antlers prior to travel, how long in advance do you do this?

57. If you have to remove antlers prior to travel, what type of restraint do you use?

Final Comments

58. Are there any areas related to the transport of deer that you feel could be improved?

59. Has the Coronavirus pandemic affected your business or how you would normally manage your deer?

If so, please explain.

60. Do you have any final comments?

61. Having participated in this study: *

I agree to the University of Bristol keeping and processing the data I have provided during the course of this study. I understand that these data will be used only for the purpose(s) set out in the information sheet, and my consent is conditional upon the University complying with its duties and obligations under the Data Protection Act.

○ I give my consent for the University to use the data that I have provided in this survey.

This content is neither created nor endorsed by Microsoft. The data you submit will be sent to the form owner.

Microsoft Forms
