# Supplementary material for: A survey of handling and transportation of UK farmed deer
Source: Anim Welf. 2023 Mar 14;32:e30. doi: 10.1017/awf.2023.25 (PMC10936360; doi:10.1017/awf.2023.25)
Supplement: Supplementary file 1 [file awfsup.zip › S0962728623000258sup002.pdf]

University of BRISTOL

Participant information sheet

What is the purpose of this study?

This study aims to assess the current state of deer transport in the UK, with a focus on transport to slaughter and its possible welfare implications. Little research has been done on the transport of deer in the UK generally, and the majority of this was done over two decades ago.

This study is split into two parts: the first part is this survey which will help us understand more about the reasons for transporting deer and how this is done; and the second part involves working with abattoirs to collect and describe data such as average journey length or average time spent in lairage and comparing this with carcass bruising data to see if there is any association. We hope that an updated understanding of how and why deer are transported in the UK will provide a basis for future research into the impact of transport on deer welfare and how this may be improved for the benefit of both the deer, and those transporting them.

Why have I been invited to participate?

As someone that is involved in the deer industry your experience and knowledge of keeping and transporting deer will help us assess the current methods for transporting deer in the UK and how this could possibly be improved.

Do I have to take part?

No, participation is voluntary and you are free to withdraw at any time, without giving a reason. If you decide you would like to withdraw after having completed the survey, please email sp16767@bristol.ac.uk requesting to withdraw your data and quote the date that you completed the survey and the first part of your postcode (as entered in the survey).

What will happen if I take part?

If after reading through this information, you decide to participate, you will be asked to give consent and then presented with the survey. The survey consists of five distinct parts: general information related to your premise, information related to transporting deer over short distances/in the local area, transport of deer over longer distances, transport of deer to slaughter and some questions related to transport in general (not specific to any distance or destination). There is also space at the end if you would like to add any comments - these could be about this survey or about the transport of deer if you feel there is something else that we should know but is not covered.

The survey should take around 10 minutes to complete. Most questions are multiple choice, with some questions having multiple answers. A few open-ended questions allow for you to comment on your experiences. You may be asked similar questions multiple times. Unfortunately, this is the only way for us to capture the information we need whilst keeping the survey simple and easy to follow, so we do apologise if this appears frustrating.

What are the possible disadvantages of taking part in the study?

There are no risks in taking part in this study other than the inconvenience of having to complete the survey in your free time.

What are the possible benefits of taking part?

By taking part in this study you will be contributing to a better scientific understanding of the transport of deer in the UK, its effect on their welfare and also, consequently, on ultimate product quality and consumer perception. A better understanding will help to focus future research projects with the aim to improve both the welfare of the deer during transport and to improve the experience for deer herd owners.

Will my participation in the project be kept confidential?

All data collected from this study will be entirely anonymous. The only personal information that we ask for is the first half of your postcode which will only be used to see how results may vary based on geographic location.

What will happen to the results of the study?

The results will be presented at a scientific meeting and we will also aim to publish the findings in a scientific journal.

Who is organising and funding the research?

This research has been funded by the charity, the Humane Slaughter Association and is being carried out at the University of Bristol

Who has reviewed the study?

This study has been reviewed by veterinarians and academics at the University of Bristol Veterinary School and also by the University of Bristol Ethics Committee.

Who can I contact if I have any queries about the survey or the research project?

Please contact Samuel Pearce, the veterinary student conducting the research, at sp16767@bristol.ac.uk
